# Patterning the spine

**DOI:** 10.7554/eLife.37288

**Published:** 2018-05-16

**Authors:** Matthew P Harris, Gloria Arratia

**Affiliations:** 1 Department of GeneticsHarvard Medical SchoolBostonUnited States; 2 Department of OrthopedicsBoston Children’s HospitalBostonUnited States; 3 Department of Systematics and Evolutionary BiologyUniversity of KansasLawrenceUnited States

**Keywords:** Notochord, segmentation clock, somite, Entpd5, evolution, vertebrae, Zebrafish

## Abstract

The patterning of the spine of a zebrafish is controlled by the notochord, a rod-like structure that supports and instructs the developing embryo.

**Related research article** Lleras-Forero L, Narayanan R, Huitema LFA, VanBergen M, Apschner A, Peterson-Maduro J, Logister I, Valentin G, Morelli LG, Oates A, Schulte-Merker S. 2018. Segmentation of the zebrafish axial skeleton relies on notochord sheath cells and not on the segmentation clock. *eLife* **7**:e33843. doi: 10.7554/eLife.33843

Patterns excite biologists. From the stripes of a zebra to the architecture of a skeleton, patterns have occupied the imagination of scientists since Aristotle and help us to understand how organisms develop and, when in disarray, why things sometimes go wrong.

The spine, for example, is a key feature of all vertebrates. It is made up of small bone units, the vertebrae, which are stacked up in an S-shaped fashion in humans. It develops from the middle germ layer of an early embryo, called the mesoderm. Some mesodermal cells go on to form a structure called the notochord, which extends from the base of the head and gives support to the developing embryo and instructs the formation of other tissues. Other cells of the mesoderm give rise to repeated segments, or somites, on either side of the head-to-tail axis. These are important to create the patterned shape of an organism and help to form the spine and other segmented structures.

One of the fundamental discoveries that led to our understanding of how such pattern arise is that the repeated units of the somites form through an oscillating on and off signal as the length of an embryo is forming, guided by a network of genes known as the segmentation clock (Pourquié, 2003). Segmentation is critical as it allows that independent parts form in specific areas: for example, our ribs are only attached to the vertebrae in our chest region, while the ribs of a snake run along their entire spine. It also increases the flexibility of the spinal column. The periodic pattern of the segmentation clock is deeply rooted in our evolutionary history, as evidenced by common developmental mechanisms shared among diverse vertebrates. Now, in eLife, Stefan Schulte-Merker and colleagues – including Laura Lleras-Forero as first author – report an oddity in fish species: the patterning of vertebrae and the formation of somites appear to be controlled by separate mechanisms in zebrafish (Lleras-Forero et al., 2018).

Previous work has shown that in some fish, the early formation of vertebrae is dependent on the notochord rather than the somites. In these species, the vertebrae emerging from the notochord develop a mineralized structure in the notochordal sheath, called the chordacentrum (Figure 1A). When the cells of the notochord are damaged, the chordacentra cannot form properly (Fleming et al., 2004). However, when the formation of somites is disrupted, the vertebrae remain normally patterned (see Fleming et al., 2015 for a review). But, when one of the key components of the central segmentation clock is altered, a dependency on somites remains, as fewer vertebrae are formed (Schröter and Oates, 2010). Such discrepancies are great opportunities to discover new aspects of how the pattern is regulated.

**Figure 1.**
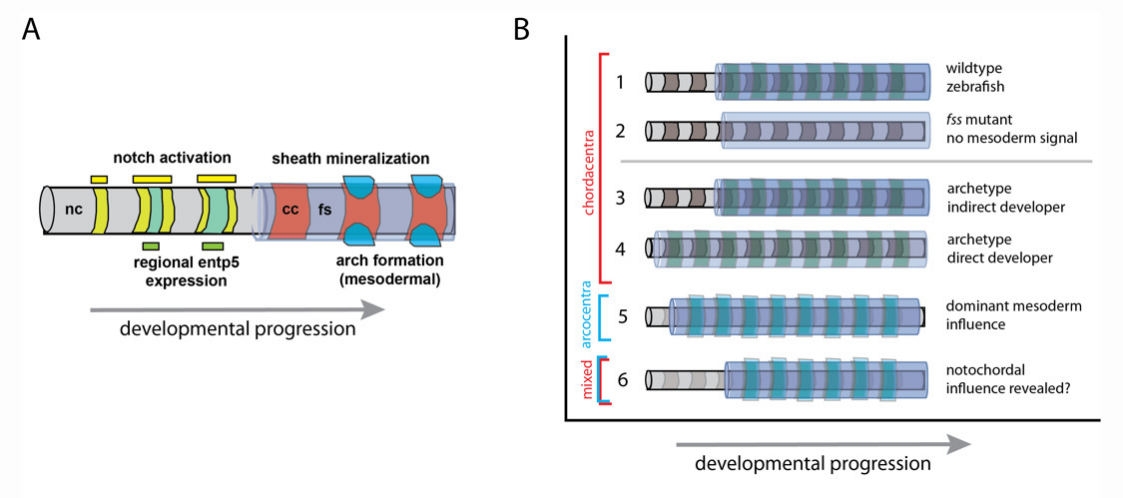
Model of vertebral patterning and variation. (**A**) Notochord patterning in the zebrafish as development proceeds, based on the research of Lleras-Forero et al. and Wopat et al. (Lleras-Forero et al., 2018; Wopat et al., 2018). The Notch signaling pathway is the earliest known signal that induces a pattern in the notochord (nc; yellow) along the head to tail axis. This activates the gene *entp5* (green) in specific domains, which in turn, leads to the mineralization of the fibrous notochordal sheath (fs; lavender) that forms the chordacentra (cc; red). The formation of arches of vertebrae (bright blue) from mesodermal cells is sensitive to – but not dependent on – the notochord pattern. (**B**) Model of notochord and mesodermal sources of vertebral pattern: (1) Patterned mineralization of the chordacentrum around notochord (nc; grey) with somite pattern (green) as seen in zebrafish. (2) Inductive role of the notochord when somite patterning is inhibited, e.g. in fish with defective segmentation clock mechanisms of the somites. (3-6) Hypotheses on variation in developmental timing and the retention of notochord patterning. (3) Indirect development as shown in the zebrafish. (4) Direct development strategies (e.g. Woltering et al., 2018), which have a contemporary activation of pattern mechanisms; here a somite signal masks a putative influence of the notochord. (5) In tetrapod vertebrates, in which the influence of the notochord is reduced or masked, the vertebrae arise from somites, and cartilage-derived tissue forms an arcocentrum (light blue). (6) Indirectly developing tetrapods, such as some salamanders, can have both mixed mineralized perichordal tissue and arcocentra (e.g., Slijepčević et al., 2018).

Lleras-Forero et al. – who are based at the University of Münster and institutes in Argentina, Germany, Switzerland, the Netherlands and the UK – introduced mutations into various genes belonging to the segmentation clock in zebrafish in order to hamper the segmentation mechanisms. Despite adding these mutations together, the chordacentra generally formed normally, although some were slightly deformed (Figure 1B). This suggests that although segmentation is regulated by the notochord, it may either be error-prone in the absence of a functional segmentation clock, or still be sensitive to signals from the overlying mesoderm.

To investigate this further, Lleras-Forero et al. tracked the activation pattern of an early cue to bone formation, a gene in the notochord sheath called *entpd5*. In normally developing zebrafish, the activation of this gene occurred in a wave-like motion from the front to the back (Figure 1A). However, in mutant fish, the waves were out of sync and gaps arose between normally patterned elements; sometimes these gaps were filled incorrectly. Computer simulations further indicated that the process does not require a clock mechanism in the somites but may arise from a separate segmentation mechanism specific to the notochord.

In related work, Michel Bagnat of Duke University and co-workers looked more closely into how the segmentation within the notochord of the zebrafish is regulated on a molecular level (Wopat et al., 2018). This work revealed that this pattern is controlled by cells in the notochord, which signal to the peripheral notochordal sheath to start the mineralization process. This signaling turned out to be dependent on a key regulator in the segmentation machinery of the somites (called Notch) that activates the *entpd5* gene (Figure 1A; Holley et al., 2002; Jiang et al., 2000). So, although different signaling properties appear to influence the segmental patterning from the notochord, they nevertheless share common signaling agents with the clock system.

A lasting question is whether the mechanism of notochord patterning is an ancient property shared by all vertebrates or, alternatively, a newer feature specific to some fish. In many fish, including zebrafish, the formation of the chordacentrum – as far as it is known – is associated with the notochord. However, basal fish, such as the lungfish, sturgeon and the coelacanth, all have persistent notochords but lack chordacentra (Arratia et al., 2001). Considering that the notochord in four-limbed vertebrates (tetrapods) has inductive and specific signaling functions necessary for the embryo and its spine to develop properly (Watterson et al., 1954), it is attractive to think that the notochord may have retained intrinsic segmentation potential over the course of evolution. Further supporting this notion, mice with disrupted somite formation can retain vertebrae segmentation, much like the mutant zebrafish (Burgess et al., 1996).

One possible explanation to the different structures formed in evolution may involve the timing of vertebrae formation (Figure 1B). Many ‘direct-developing’ tetrapods depend on long-lasting nutrition from their mother during their early development. In contrast, some animals, including fish, already feed independently as larvae when their adult characteristics have not yet completely formed. Such ‘indirect’ development can arise when developmental programs are activated at different times. These different 'developmental strategies’ can reveal unique aspects of development that might not normally be seen.

It may be that in many vertebrates, although present, notochord patterning is masked by a dominant somite developmental program (Figure 1B, model 4, as proposed in Fleming et al., 2015). This would be in agreement with the findings of Lleras-Forero et al., which showed potential interaction between the notochord and somites, reinforcing the pattern of the forming vertebral components. Work in ‘indirect’ developing salamanders and ‘direct’ developing fish give support to this proposition (Figure 1B; Slijepčević et al., 2018; Woltering et al., 2018). By this model, the early formation of the mineralized, segmented notochord in zebrafish may dissociate, or even expose, these two patterning mechanisms.

Damage to the lasting derivatives of the notochord, such as the discs and joints between the vertebrae, are a leading cause of back pain associated with many diseases and aging (Dowdell et al., 2017). It would be interesting to see if alteration in such patterning processes in the notochord could explain a tendency for skeletal problems later in life. Could the zebrafish be an ideal model to address these questions? Nevertheless, the uncovering of an autonomous role for the notochord to create segmentation and patterns excites our imagination of how it arises during development and how it changes during evolution.

## References

[bib1] Arratia G, Schultze HP, Casciotta J (2001). Vertebral column and associated elements in dipnoans and comparison with other fishes: development and homology. Journal of Morphology.

[bib2] Burgess R, Rawls A, Brown D, Bradley A, Olson EN (1996). Requirement of the paraxis gene for somite formation and musculoskeletal patterning. Nature.

[bib3] Dowdell J, Erwin M, Choma T, Vaccaro A, Iatridis J, Cho SK (2017). Intervertebral disk degeneration and repair. Neurosurgery.

[bib4] Fleming A, Keynes R, Tannahill D (2004). A central role for the notochord in vertebral patterning. Development.

[bib5] Fleming A, Kishida MG, Kimmel CB, Keynes RJ (2015). Building the backbone: the development and evolution of vertebral patterning. Development.

[bib6] Holley SA, Jülich D, Rauch GJ, Geisler R, Nüsslein-Volhard C (2002). *her1* and the *notch* pathway function within the oscillator mechanism that regulates zebrafish somitogenesis. Development.

[bib7] Jiang YJ, Aerne BL, Smithers L, Haddon C, Ish-Horowicz D, Lewis J (2000). Notch signalling and the synchronization of the somite segmentation clock. Nature.

[bib8] Lleras-Forero L, Narayanan R, Huitema LFA, VanBergen M, Apschner A, Peterson-Maduro J, Logister I, Valentin G, Morelli LG, Oates A, Schulte-Merker S (2018). Segmentation of the zebrafish axial skeleton relies on notochord sheath cells and not on the segmentation clock. eLife.

[bib9] Pourquié O (2003). The segmentation clock: converting embryonic time into spatial pattern. Science.

[bib10] Schröter C, Oates AC (2010). Segment number and axial identity in a segmentation clock period mutant. Current Biology.

[bib11] Slijepčević MD, Ukropina M, Filipović B, Ivanović A (2018). Ossification and development of vertebrae in the Balkan crested newt *Triturus ivanbureschi* (Salamandridae, Caudata). Zoology.

[bib12] Watterson RL, Fowler I, Fowler BJ (1954). The role of the neural tube and notochord in development of the axial skeleton of the chick. American Journal of Anatomy.

[bib13] Woltering JM, Holzem M, Schneider RF, Nanos V, Meyer A (2018). The skeletal ontogeny of *Astatotilapia burtoni* – a direct-developing model system for the evolution and development of the teleost body plan. BMC Developmental Biology.

[bib14] Wopat S, Bagwell J, Sumigray KD, Dickson AL, Huitema LFA, Poss KD, Schulte-Merker S, Bagnat M (2018). Spine patterning is guided by segmentation of the notochord sheath. Cell Reports.

